# Interleukin-32: a Pandora’s box in human immunodeficiency virus infection

**DOI:** 10.3389/fimmu.2025.1708955

**Published:** 2025-11-26

**Authors:** Abualgasim Elgaili Abdalla, Emad Manni, Tilal Elsaman, Khalid Abosalif, Ayman Ali Mohammed Alameen, Malik Suliman Mohamed, Hasan Ejaz

**Affiliations:** 1 Department of Clinical Laboratory Sciences, College of Applied Medical Sciences, Jouf University, Sakaka, Saudi Arabia; 2 Department of Pharmaceutical Chemistry, College of Pharmacy, Jouf University, Sakaka, Saudi Arabia; 3 Department of Pharmaceutics, College of Pharmacy, Jouf University, Sakaka, Saudi Arabia

**Keywords:** interleukin-32, human immunodeficiency virus, immunosuppression, latency, cardiovascular disease

## Abstract

Human immunodeficiency virus (HIV), which causes acquired immunodeficiency syndrome (AIDS), has been estimated to infect around 40.8 million people globally. This virus can persist and thrive in the presence of antiretroviral therapy, which is a major obstacle to HIV eradication. Therefore, understanding host immunological factors that underlie HIV persistence and pathogenesis can lead to the development of immunotherapeutic interventions. Interleukin-32 (IL-32) is an orphan cytokine with multiple isoforms and a complex signal transduction pathway that transmits through non-specific receptors. It is a multifunctional cytokine with dual immunomodulatory roles in HIV infection. IL-32 possesses both antiviral and pathogenic properties. It can block viral entry to target cells and reverse transcriptase activity. Also, IL-32 can promote the reactivation of latent reservoirs. Paradoxically, IL-32 can inhibit HIV-specific immune response and facilitate HIV latency in CD4+ T cells. IL-32 has a central pathological role in HIV-related cardiovascular disease. Here in this review, we will discuss the biology of IL-32 and the current state-of-the-art knowledge of how IL-32 orchestrates diverse immune responses during HIV infection. In addition, the potential therapeutic strategies that could modulate IL-32 activity or expression will be highlighted.

## 1 Introduction

Acquired immunodeficiency syndrome (AIDS) has remained a global health calamity for over 42 years, claiming the lives of more than 42 million individuals worldwide. In 2024, an estimated 40.8 million individuals were living with HIV globally, with women accounting for 51.4%, men for 45.3%, and children for 3.4% of the affected population (1).

Human immunodeficiency virus (HIV) is the etiological agent of AIDS. It belongs to the genus *Lentivirus* from the *Retroviridae* family. Two divergent species, HIV-1 and HIV-2, have been identified globally (2). HIV has low-fidelity reverse transcriptase, which converts the viral RNA genome into DNA at the initial step of viral replication. This process results in extensive mutation, leading to the emergence of genetically divergent strains (3), which is a viral immune evasion strategy. HIV can hijack the immune system response by invading and replicating/or establishing latency in the target CD4+ cells, including T lymphocytes, which are principal regulators of adaptive immunity (4), and monocytes/macrophages, which are the frontline of innate cellular defense (5). HIV can induce CD4+ T cell death, leading to substantial impairment of host immune function (6). In addition, HIV can exploit macrophages to disseminate throughout the body, manipulate the host’s killing machinery, or induce an intense tissue inflammation reaction (7).

Cytokines, including interleukins (ILs), are secreted proteins that serve as immunological messengers, orchestrating the development, maturation, function, or inhibition of immune cells following engagement with their specific receptors (8). ILs are secreted not only by leukocytes, but also various nonimmune cells (9). ILs shape host immune responses during HIV infection, thereby directly affecting viral containment and clinical outcomes (10). The elucidation of how specific ILs attenuate or accelerate HIV replication has the potential to inform the design of immunotherapeutic interventions.

IL-32 is an orphan cytokine that differs from other known cytokines in structure and partner receptors. At least 10 distinct IL-32 isoforms have been identified and are expressed in multiple cell types, including leukocytes and nonimmune cells such as epithelial and endothelial cells (11–13). Functionally, the role of three isoforms, IL-32α, β, and γ, in regulating immune responses against cancer is a double-edged sword, as they exert anti-cancer effects, while also promoting cancer growth and metastasis (13, 14). Similar isoforms, IL-32θ and δ, enhance the production of pro- and anti-inflammatory mediators in a variety of autoimmune diseases (15).

In tuberculosis, the levels of IL-32β and γ are positively correlated with mycobactericidal regulator, namely interferon-gamma (IFN-γ), and negatively correlated with those of the massive inflammatory mediators, IL-6 and IL-17 (16). No homology of IL-32 has been identified in mice. However, mice respond to IL-32, and therefore, the biological functions of IL-32 have been investigated using transgenic mice (17, 18). It was shown that IL-32γ transgenic mice displayed better control of *Leishmania braziliensis* and improved healing of cutaneous lesions, compared to wild-type mice (18). The presence of IL-32γ leads to improved hepatitis B virus clearance due to the cytokine’s enhancement of IFN-λ1 expression both ex vivo and *in vivo* (19). However, IL-32, especially the most extensively studied IL-32γ, has a Pandora’s box of roles in HIV infection, as it drives antiviral immunity, impairs the antiviral immune control, and mediates HIV-related cardiovascular diseases (20–23).

Therefore, IL-32 is a necessary conductor of host immune responses. An understanding of the molecular regulatory mechanisms of IL-32 expression and the IL-32 signaling that shapes the host immune responses is crucial for the development of immune-directed therapies for various diseases. This review will cover signal-transduction and epigenetic regulation of *IL32* expression, as well as IL-32 signaling mechanisms. The main scope of the review is to discuss the current state-of-the-art knowledge of how IL-32 orchestrates diverse immune responses during HIV infection. In addition, the potential therapeutic strategies that could modulate IL-32 activity or expression will be highlighted.

## 2 Molecular features and regulation of IL-32 expression

IL-32 is a distinct inflammatory mediator, first described as NK transcript 4 (NK4) due to its significant upregulation of natural killer (NK) cells following IL-2 stimulation (24). The *IL32* gene is localized on chromosome 16p13.3 and comprises eight exons. These exons undergo posttranscriptional alternative splicing, giving rise to multiple mRNA transcripts that encode different protein isoforms (11). To date, at least 10 IL-32 isoforms have been identified, designated as IL-32α, IL-32β, IL-32γ, IL-32δ, IL-32ϵ, IL-32ζ, IL-32η, IL-32θ, and IL-32s, as well as IL-32D, which are diverse in size, structure, and biological activity (25–27). IL-32D has recent identified as proinflammatory mediator and 95% of its amino acid sequence similar to that of IL-32β (26). The regulation of IL-32 isoform expression, including factors that govern splicing events and posttranscriptional modifications, is not yet fully understood. Further studies are needed to elucidate how these processes contribute to cell-type-specific expression patterns and functional diversity. Due to the absence of antibodies specific to each isoform, studies on IL-32 regulation have largely depended on analysis of mRNA expression for each variant and measurement of total protein levels of IL-32 (28, 29).

Of the IL-32 isoforms identified to date, seven primary variants (α–θ) have been extensively studied and successfully cloned and purified. The longest isoform, IL-32γ (234 amino acids), contains the full complement of 11 putative protein domains, whereas IL-32α, the shortest isoform (131 amino acids), includes only domains 1, 6, and 11. Other isoforms, such as IL-32β (188 residues), IL-32δ (178 residues), IL-32ϵ (148 residues), IL-32ζ (179 residues), and IL-32θ (168 residues), are intermediate in length and domain composition (30). Although these domain assignments are based on sequence prediction and functional mapping, the complete biological significance of each domain remains under investigation.

To date, experimentally determined three-dimensional (3D) structures of IL-32 isoforms have not been reported in the Protein Data Bank. This limitation is largely attributed to the absence of signal peptide in all IL-32 isoforms with the exception of IL-32γ, thereby resulting in their low quantities in cell culture supernatants and posing significant difficulties for protein purification (31). Computational modelling approaches, particularly using I-TASSER software, have been employed to predict the secondary and tertiary structures of several IL-32 isoforms (32). These models suggest that IL-32α and IL-32β adopt a helical-bundle architecture, primarily composed of α-helices and random coils, with an apparent lack of β-sheets. The modelling of IL-32γ has proven to be more challenging due to its longer sequence and the predicted presence of a transmembrane-like region, which complicates in silico folding predictions (32). Further experimental efforts, such as cryo-electron microscopy or nuclear magnetic resonance spectroscopy, are needed to clarify its full 3D conformation. All major IL-32 isoforms share a conserved arginine–glycine–aspartate (RGD) motif, known to interact with integrins and potentially mediate intracellular signaling or adhesion-related functions. Interestingly, mutation of the RGD motif does not inhibit IL-32β or IL-32γ signaling, implying that other structural regions or interacting partners may drive their activities (30, 32). Also, the structural modelling of IL-32 predicts the presence of the DDM motif, which may participate in IL-32 interaction with the integrin signaling components. This motif may also contribute to isoform–isoform interactions, thereby shaping regulatory dynamics among IL-32 variants (32). However, the role of DDM motifs in the biological function of IL-32 requires experimental verification. Given the emerging role of IL-32 variants in diverse pathological processes, including inflammatory conditions, infectious diseases, and immune imbalances in HIV infection, the elucidation of IL-32 (3D) structures and domain interactions has become a high priority. Molecular insights into IL-32 may advance the rational design of therapeutics that target IL-32-driven signaling pathways.

**Figure 1:**
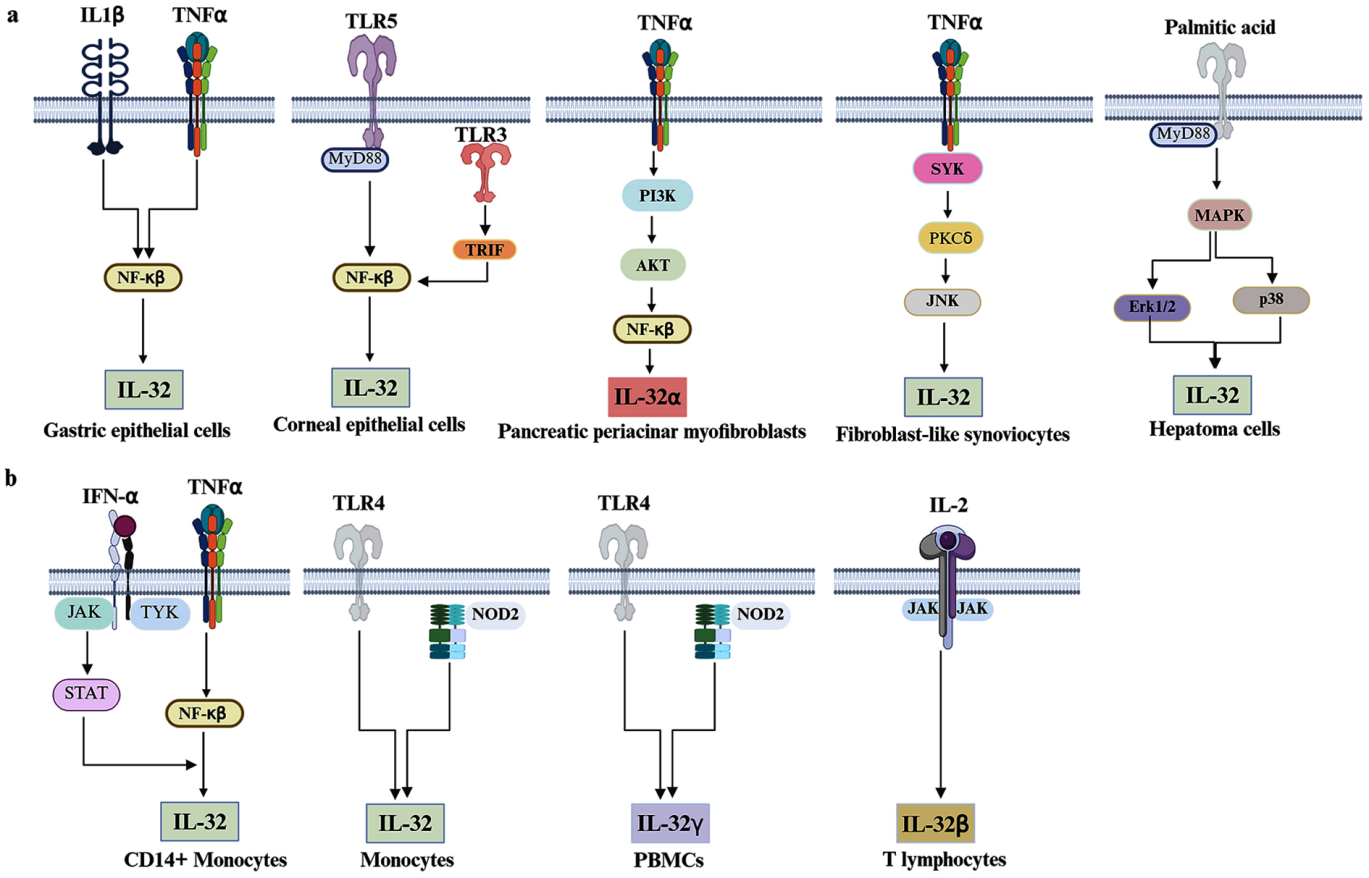
Regulatory pathways governing IL-32 expression. **(a)** IL-32 expression in nonimmune cells is regulated by a variety of signaling cascades, including TNF-α, TLRs, and IL-1β. These activate NF-κB, which is a key regulator of IL-32 expression in gastric and corneal epithelial cells. In pancreatic periacinar myofibroblasts, TNF-α enhances IL-32α expression through activation of the NF-κB signaling cascade. In addition, TNF-α promotes IL-32 expression in fibroblast-like synoviocytes via the SYK/PKCδ/JNK axis, whereas in hepatoma cells, IL-32 transcription is positively upregulated by the MAPK cascade (ERK and p38) following palmitic acid stimulation. **(b)** The regulatory landscape in hematopoietic cells, including monocytes, T lymphocytes, and peripheral blood mononuclear cells (PBMCs). In this compartment, IL-32 expression is enhanced in CD14+ monocytes by the synergistic action of TNF-α and IFN-α, which activate NF-κB and STAT, respectively, while NOD2 and TLR4 signaling induce IL-32 and IL-32γ expression in monocytes and PBMCs, respectively. IL-32β expression in T cells is regulated by IL-2R signaling. IL-32 in a pale green box indicates naïve IL-32.

IL-32 is known to be expressed in both non-hematopoietic and hematopoietic cells (**Figure 1**). In non-hematopoietic cells, IL-32 expression is upregulated in hepatoma cells (Huh7.5) (33), human aortic valve interstitial cells (34), and the human gastric epithelial cell line (35). Proinflammatory cytokines, such as tumor necrosis factor-alpha (TNF-α) and IL-1β (35), and various toll-like receptors (TLRs) contribute to the induction of IL-32 transcription through activation of the NF-κB signaling pathway (36). Pharmacological inhibitors of NF-κB, such as BAY 11–7082 and MG132, significantly downregulate IL-32 transcription and diminish its protein production (37, 38). Similarly, targeting suppression of NF-κB signaling through a small molecule IKKβ inhibitor SC-514 or by IKKβ-specific siRNA, leading to marked decreases in IL-32 transcription levels in gastric epithelial cells challenged with *Helicobacter pylori* (39). In human pancreatic periacinar myofibroblasts (HPPMs), TNF-α can induce IL-32α expression through the activation of the phosphoinositide-3-kinase (PI3K)/Akt pathway, which subsequently triggers NF-kβ signaling (40). However, TNF-α enhances IL-32 transcription in fibroblast-like synoviocytes (FLS) through the activation of tyrosine-protein kinase (SYK), protein kinase C delta (PKCδ), and c-Jun N-terminal kinase (JNK) signaling cascades (41). TNF-α and interferon-gamma (IFN-γ) trigger epithelial cell death, leading to the liberation of IL-32 via a nonconventional secretory pathway involving exosomes and vesicular bodies. Under these conditions, in culture supernatant, IL-32 has been detected bound with lipid droplets (42).

Extracellular signal-regulated kinase (ERK) and p38, integral elements of the mitogen-activated protein kinase (MAPK) cascade, are critically involved in modulating the production of proinflammatory mediators in response to cellular stress (43). Stimulation of hepatoma cell lines with palmitic acid induces cellular stress and IL-32 expression by activating the ERK and p38-MAPK cascade (44). Nevertheless, in both FLSs and HPPMs, the MAPK pathway is not part of the signalome that mediates IL-32 transcription (40, 41).

In hematopoietic cell types, TNF-α and IFN-α act synergistically to upregulate expression and secretion of IL-32 by CD14+ monocytes through the activation of NF-kβ and Janus kinase/signal transducer and activator of transcription (JAK/STAT) signaling, respectively (37). The triggering of monocytes with TLR4 and nucleotide-binding oligomerization domain-containing protein 2 (NOD2) agonist increases IL-32 transcription (45). These two receptors are also required to induce IL-32γ secretion by peripheral blood mononuclear cells (PBMCs) following stimulation with lipophosphoglycan derived from *Leishmania* species (46). The specific downstream transcription factors mediating IL-32 transcription in response to TLR4 and NOD2 signaling have yet to be fully identified.

Three IL-32 variants, IL-32α, IL-32β, and IL-32γ transcripts, have been found at significantly higher levels in pleural fluid mononuclear cells from patients with tuberculosis, compared to their expression in PBMCs from the same patients and healthy controls. Within this cellular milieu, monocytes/macrophages serve as the principal sources of IL-32. However, cytosolic IL-32 has also been demonstrated in CD4+ helper and CD8+ cytotoxic T lymphocytes (47). The dynamic cross-talk between monocytes/macrophages and lymphocytes appears to be a key regulatory mechanism for the induction of IL-32 expression (47). Nevertheless, the specific receptors and transcriptional regulators orchestrating this process have yet to be delineated.

T cells have emerged as the principal source of IL-32 under diverse pathological conditions. The expression and extracellular release of the IL-32β isoform by T cells are governed by distinct signaling axes as follows: IL-2 signaling upregulates IL-32β expression, whereas T cell receptor (TCR) signaling mediates extracellular release of this isoform. Mechanistically, TCR activation leads to the formation of membrane pores, permitting the extracellular delivery of IL-32β (48). However, studies of multiple myeloma cell lines show that IL-32γ is exported to the extracellular milieu through incorporation into exosomes (49). Taken together, although significant progress has been made in elucidating the regulation of IL-32 expression in various biological models, several key questions remain unanswered. Future research should address how IL-32 isoforms are differentially regulated in distinct cell types, whether splice site mutations influence isoform production, and the mechanisms underlying IL-32 secretion. In addition, it is crucial to determine whether all IL-32 isoforms are exported extracellularly by the same pathway or whether isoform-specific differences exist.

## 3 Epigenetic regulation of IL-32 expression

**Figure 2:**
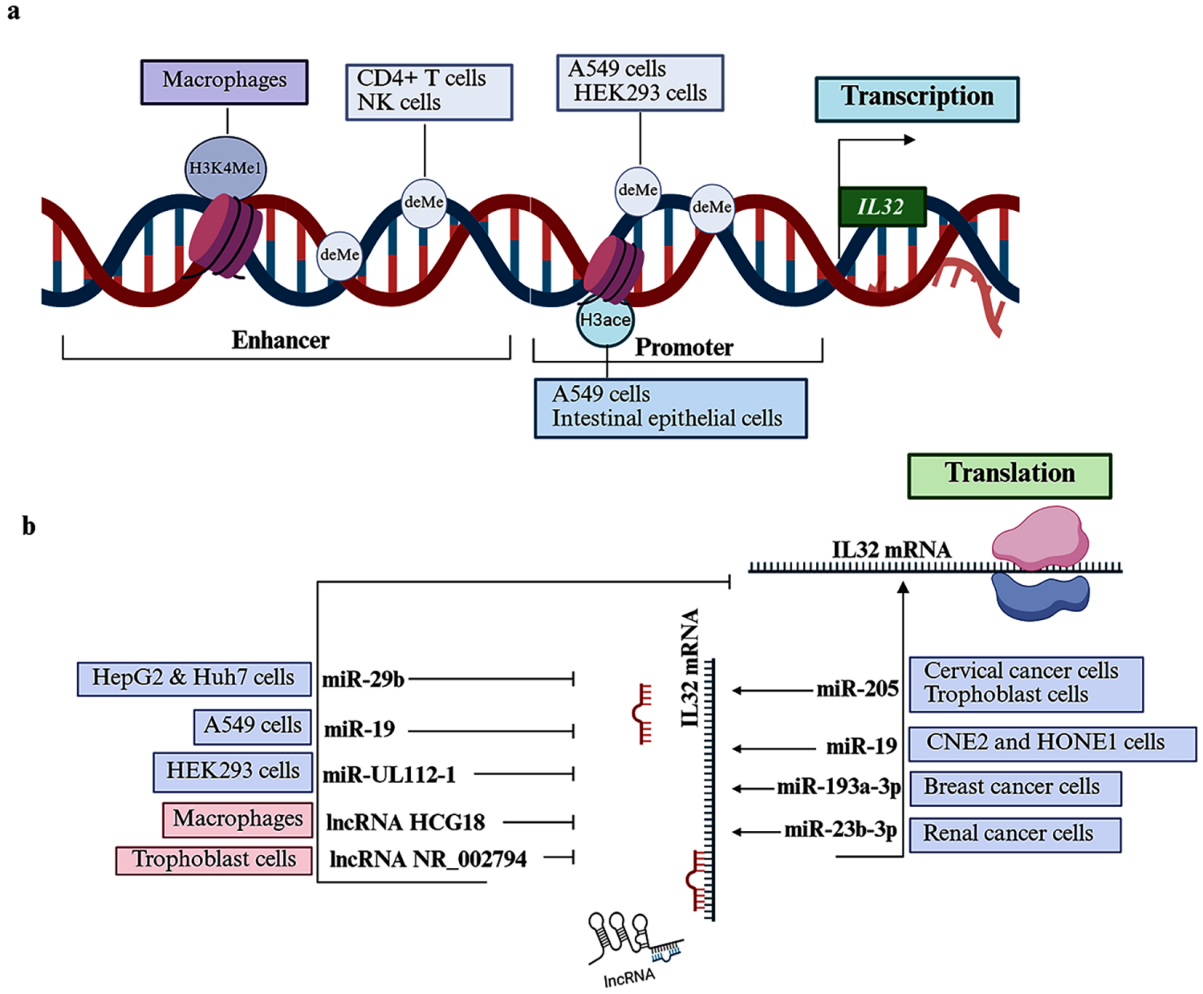
Epigenetic regulation of IL32 expression. **(a)** IL-32 transcription is modulated by various epigenetic mechanisms. In macrophages, monomethylating histone H3 lysine 4 (H3K4me1) at the enhancer element increases IL-32 expression. In CD4+ T cells and natural killer (NK) cells, DNA demethylation (deMe) within the enhancer region is essential for IL-32 transcription activation. Similarly, DNA demethylation at the promoter region enhances IL-32 transcription in lung epithelial cells (A549 cells) and human embryonic kidney-293 cells (HEK293 cells). In intestinal epithelial cells, histone 3 acetylation (H3ace) at the promoter promotes IL-32 expression, whereas deacetylation of histone H3 at the promoter is associated with the upregulation of IL-32 expression in A549 cells. **(b)** IL-32 expression is regulated by diverse noncoding RNAs. microRNAs (miRs), such as miR-29b, miR-19, miR-UL112-1, and long noncoding RNAs (lncRNAs), such as lncRNA-HCG18 and lncR NR_002794, act as negative regulators of IL-32 expression in HepG2 and Huh7 cells, A549 cells, HEK293 cells, macrophages, and trophoblast cells, respectively. Conversely, miR-205, miR-19, miR-193a-3p, and miR-23b-3p have been shown to promote IL-32 expression in cervical cancer cells and trophoblast cells, nasopharyngeal carcinoma cell lines (CNE2 and HONE1 cells), breast cancer cells, and renal cancer cells, respectively.

The epigenetic process plays a central role in regulating IL-32 expression, chiefly involving DNA methylation, histone acetylation (**Figure 2a**), and noncoding RNAs (**Figure 2b**). Hypomethylation of the *IL32* gene has been identified as an epigenomic signature in CD4+ T cells from patients with juvenile idiopathic arthritis, indicating a potential contribution to the disease (50). An *in vitro* assay confirmed that methylation at the promoter and upstream regions of *IL32* leads to decreased gene expression (51). Further multiomics analysis of CD4+ T cells and NK cells showed that increased IL-32 transcription is directly linked with the DNMT3-mediated demethylation state at the enhancer site (52). DNA demethylation at the IL-32 promoter has also been shown to upregulate IL-32 expression in A549 cells infected with the influenza virus (38). In particular, demethylation at the nucleotide site (–24 to –17) enables transcription factor CREB binding, thereby enhancing IL-32 expression (38). Inflammatory stimuli, such as TNF-α, can also induce persistent DNA demethylation at the IL-32 promoter region, thereby instigating IL-32 expression in human embryonic kidney-293 (HEK293) cells (53). This hypomethylated state is indispensable for IL-32 transcription, as it facilitates the binding of NF-kβ to the upstream regulatory region of the *IL32* gene (53). Moreover, the monomethylated histone H3 lysine 4 (H3K4me1) at the enhancer element significantly upregulates the transcription of IL-32 in β-glucan-activated macrophages (54).

The binding of transcription factors to the IL-32 promoter is enhanced by histone (H3) hyperacetylation, which is correlated with amplified IL-32α transcription in IL-1β-activated human intestinal epithelial cell lines (55). However, an *in vitro* study demonstrated that IL-32 expression is significantly downregulated in A549 cells following treatment with pharmacological inhibitors of histone deacetylase (56). The cell-type-specific mechanisms by which histone acetylation modulates differential IL-32 remain incompletely characterized.

Noncoding RNAs, including microRNAs (miRs) and long noncoding RNAs (lncRNAs), are another level of epigenetic regulators of gene expression. miRs can directly affect the translation of IL-32 via posttranscriptional targeting of mRNA. For example, miR-29b can directly target the 3’-UTR of IL-32 and reduce its level through acceleration of IL-32 mRNA degradation in HepG2 and Huh7 cells on infection with the hepatitis B virus (19). miR-205 also regulates IL-32 expression in multiple cell lines, including trophoblast and human cervical cancer cell lines. This miR can increase the expression level of IL-32 by directly targeting the IL-32 regulatory region (57, 58). Knockout of miR-23b-3p has been found to downregulate the expression of IL-32 in A-498, a renal cancer cell line (59). Ectopically expressed miR-193a-3p in breast cancer cells (MCF-7) resulted in positive upregulation of IL-32 expression (60). However, the underlying molecular mechanisms by which miR-23b-3p and miR193a-3p regulate IL-32 expression need to be determined.

The forced miR-19 expression markedly reduces IL32 levels in lung epithelial cells, including A549 and HCC827 cell lines, while it can increase IL-32 expression in nasopharyngeal epithelial cancer cell models, including CNE2 and HONE1 cells (61). The molecular pathway targeted by miR-19 to modulate IL-32 expression in a cell-type-specific manner needs to be examined. Interestingly, human cytomegalovirus (HCMV)-encoded miRs, namely, miR-UL112-1, can also reduce both mRNA and IL-32 protein levels in HEK293 by targeting the 3’-UTR of IL-32 mRNA following HCMV infection (62).

A recent study on the role of exosome-mediated acute lung injury demonstrates that lncRNA HCG18 is crucial for the regulation of IL-32 expression during exosomal communication between polymorphonuclear cells (PMNs) and macrophages. Mechanistically, PMNs release exosomes containing HCG18, which are subsequently taken up by macrophages. lncRNA HCG18 can impede IL-32 expression in macrophages and, therefore, enhance the polarization of macrophages into inflammatory (M1) cells (63). lncRNA NR_002794 can suppress IL-32 expression in human trophoblast cells via downregulation of Akt and the ERK1/2 MAPK pathway (64).

## 4 IL-32 signaling mechanisms

Although extensive research has been conducted to decipher the functions of IL-32 in different pathological conditions, the specific binding partner of IL-32 remains largely undetermined. Some studies suggest that IL-32 induces immune-regulatory activities through engagement with nonclassical receptors, which are quite different from those used by other interleukins (13, 30). These include surface and intracellular partners receptors, such as proteinase (PR3) (28, 65, 66), integrins (32, 67), as well as intracellular receptors such as protein kinase C (PKC) (68–70) and focal adhesion kinase (FAK) (32, 71).

**Figure 3:**
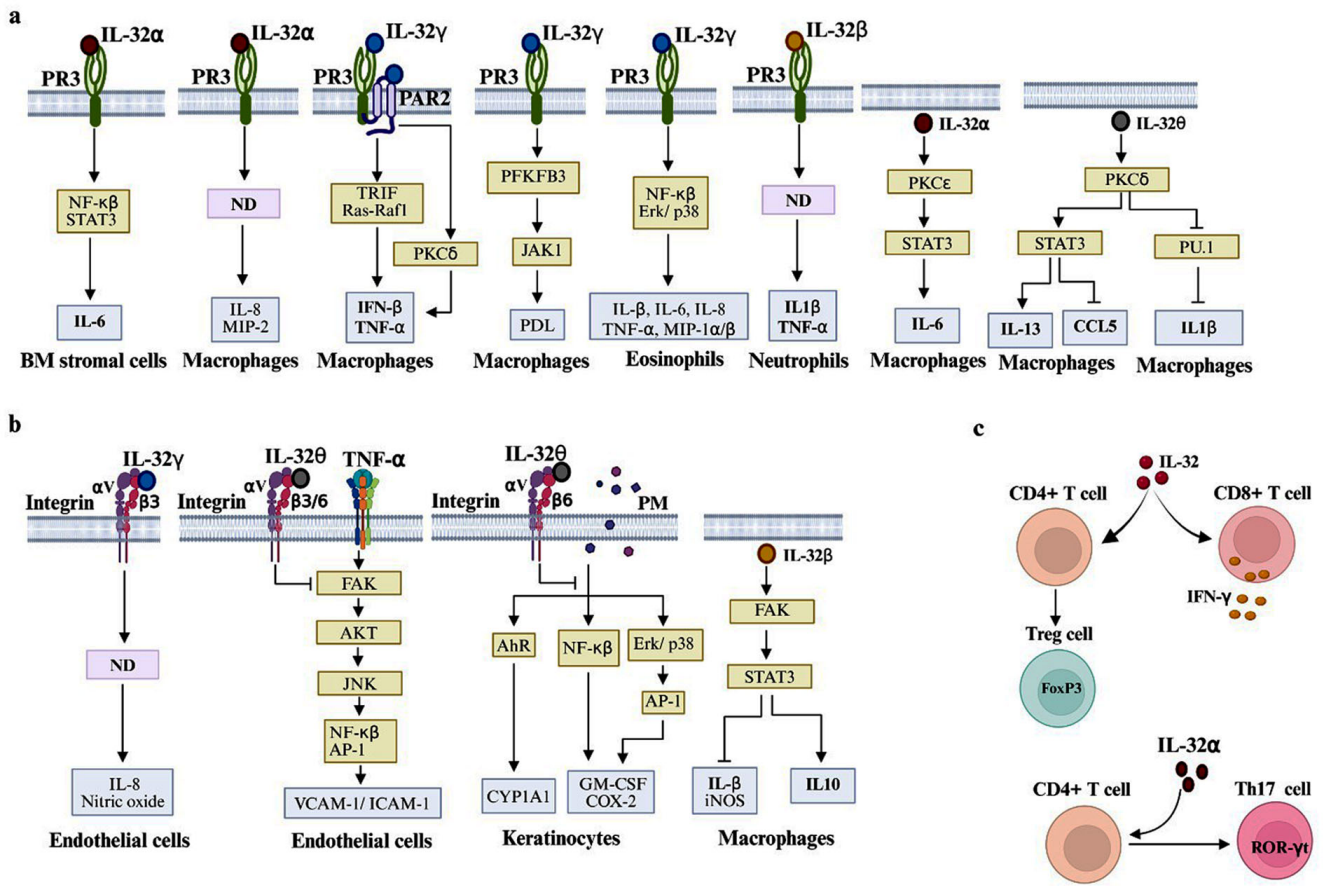
IL-32 signaling pathways modulate immune and nonimmune cell responses. **(a)** IL-32 orchestrates a spectrum of signaling events through interactions with PR3, PAR2, and PKC, thereby modulating cytokine production by myeloid lineage cells and bone marrow (BM) stromal cells. **(b)** IL-32’s interaction with integrins directly influences the biological activities of nonimmune cells, such as endothelial cells and keratinocytes. In macrophages, IL-32β can directly activate the FAK-STAT3 pathway, leading to the polarization of macrophages into anti-inflammatory phenotypes. **(c)** IL-32 exerts a dual regulatory role in lymphocyte function by promoting the production of IFNγ by CD8+ T cells, while also driving CD4+ T cell polarization toward a regulatory (Treg) phenotype. However, IL-32α induces helper T cell differentiation into the Th17 subset. ND, not determined; PM, particulate matter.

Proteinase 3 (PR3), a membrane-bound or secretory neutral serine protease that is expressed across various cell types, such as myeloid lineage cells and epithelial cells (72, 73), modulates host immune responses (74). IL-32α’s interaction with PR3 consistently results in the production of chemotactic factors, such as macrophage inflammatory protein-2 (MIP-2) and IL-8 by mouse and human macrophages, respectively (65). In addition, the interaction between IL-32α and PR3 enhances IL-6 production by bone marrow stromal cells through the induction of NF-κB and STAT3 activation (**Figure 3a**) (66).

In THP-1 macrophages, the binding of IL-32γ and PR3 has been found to trigger the protease-activated receptor 2 (PAR2) cascade (**Figure 3a**) (75). This signaling subsequently induces the transcription of IFN-β and TNF-α through the activation of TIR domain-containing adaptor-inducing interferon-β and Ras-Raf1 signaling. Protein kinase C (PKC) isoforms δ and ϵ are involved in IL-32γ-PAR2 signaling, as indicated by the dampening of PKCδ resulting in reduced IL-32γ-regulated expression of IFN-β and TNF-α, while PKCϵ has the opposite effect (**Figure 3a**) (75). Overexpression of IL-32θ in THP-1 macrophages can enhance PKCδ activity, which blocks PU.1 transcription factor phosphorylation and subsequently diminishes IL-1β transcription (76). IL-32θ provokes PKCδ-dependent activation of STAT3, which subsequently suppresses inflammatory chemokine CCL5 (68) and induces anti-inflammatory cytokine IL-13 production by THP-1 macrophages (70). However, the triggering of the PKCϵ-STAT3 pathway by endogenous IL-32α results in enhanced IL-6 secretion by PMA-treated THP-1 cells (69). These findings indicate that PKCδ and PKCϵ exert differential regulatory effects on inflammatory cytokine production by macrophages, dependent on the specific IL-32 isoform stimulus (**Figure 3a**).

IL-32γ can potentiate eosinophils to secrete proinflammatory mediators, such as IL-1β, TNF-α, IL-6, IL-8, and MIP-1α/β, through the triggering of PR3 signaling activation, with a markedly amplified effect of NOD ligand stimulation. This synergistic effect in eosinophils results from the activation of NF-κB, MAPK ERK1/2, and p38 (**Figure 3a**). The same study demonstrated that IL-32α exhibits comparable activity, but less marked activity than IL-32γ (77). IL-32γ promotes PR3-dependent enhancement of 6-phosphofructo-2-kinase/fructose-2,6-biphosphatase 3 (PFKFB3) expression, subsequently triggering JAK1-mediated induction of programmed death-ligand 1 (PDL-1) in macrophages (**Figure 3a**) (49). IL-32β can intensify the inflammatory reaction by increasing the secretion of proinflammatory mediators, such as IL-1β, IL-6, TNF-α, and IL-8, by PMNs via PR3 signaling activation (**Figure 3a**) (78). The transcription factors downstream of PR3 that are activated in PMN in response to IL-32β stimulation remain to be investigated.

Integrins are transmembrane-anchored heterodimeric proteins that consist of α and β glycoprotein subunits held together by a noncovalent bond. These molecules are indispensable for cell–cell crosstalk, a sensor of the extracellular milieu, and signal transduction (79, 80). IL-32α has been found to interact with αvβ3 and αvβ6 integrin, while IL-32β and γ are also capable of interacting with αvβ3 integrin, but with lowered affinity relative to that of IL-32α. Structurally, IL-32 mimics the focal adhesion target (FAT); therefore, it can compete with FAT for the binding site at the downstream components of integrin, particularly paxillin (PAX) and focal adhesion kinase (FAK) (32). IL-32γ interferes with integrin αvβ3–mediated activation of the PAX-FAK signaling pathway in transforming growth factor-beta (TGF-β)-stimulated MRC5 cells (lung fibroblast cells), thereby blocking the production of the actin marker of fibroblast cells, namely α-smooth muscle actin (α-SMA), and adhesion molecules (fibronectin) (81). Nonetheless, IL-32γ can induce IL-8, nitric oxide, and pro-angiogenic molecule production by IFN-γ-stimulated endothelial cells by triggering the integrin αVβ3 signaling pathway (67) (**Figure 3b**), which implies that it has a role in angiogenesis.

IL-32 mediates bidirectional signaling within the tumor microenvironment through an integrin αvβ3-dependent and/or FAK-regulated pathway. Specifically, IL-32γ released by cancer-associated fibroblast cells promotes the expression of metastatic markers in breast cancer cells by integrin αvβ3 signaling–mediated activation of p38 MAPK (82). In contrast, an *in vivo* study in which CD133+ cancer stem cells (CSCs) were injected into transgenic mice expressing IL-32γ and wild-type mice showed that the transgenic mice had better control of CSCs growth than their non-transgenic counterparts. IL-32γ-integrin β3 signaling leads to the abrogation of STAT5 phosphorylation, thereby enhancing cancer cell death and arresting cell growth (83).

Elevated IL-32β expression is strongly associated with esophageal squamous cell carcinoma (ESCC) invasion of lymph nodes and metastasis. IL-32β is released from ESCC within the vesicle and is subsequently internalized by nearby macrophages. Functional analysis revealed that IL-32β promotes macrophage differentiation toward an anti-inflammatory phenotype (M2) by activation of the FAK-mediated STAT3 phosphorylation cascade. Transcriptomic analysis of these M2 macrophages showed pronounced upregulation of the anti-inflammatory mediator IL-10, accompanied by diminished expression of proinflammatory mediators, including IL-1β and inducible nitric oxide synthase (**Figure 3b**) (71).

The IL-32θ variant, which has an alanine-substituted valine at position 94 (A94V), has recently been shown to possess the ability to bind to αvβ3 and αvβ6 integrins (84). It attenuates TNF-α-induced upregulation of the adhesion molecules CD54 (ICAM-1) and CD106 (VCAM-1) in endothelial cells, thereby diminishing monocyte–endothelial cell adhesiveness. Mechanistically, this isoform suppresses the expression of adhesion molecules by inhibiting FAK-dependent phosphorylation of Akt and JNK, which in turn limits the nuclear translocation of NF-kβ components and the AP-1 transcription factor (**Figure 3b**) (84). The IL-32θ (A94V) variant engages with αvβ6 integrin on the surface of keratinocytes (HaCaT) (85). IL-32θ (A94V) can hinder particulate matter (PM)-induced production of the inflammatory mediator COX-2, GM-CSF, and cytochrome P450 enzyme (CYP1A1). Multiple signaling pathways induced by PM in HaCaT cells, including NF-κB, ERK/p38-MAPK, and aryl hydrocarbon receptor (AhR), were found to be repressed by IL-32θ (A94V)-αvβ6 integrin signaling (**Figure 3b**) (85).

IL-32 can influence the fate and function of lymphocytes. In CD8+ T cells, IL-32 promotes IFN-γ production, supporting robust antitumor and antimicrobial responses. In CD4+ T cells, IL-32 enhances the development of regulatory T (Treg) cells by Foxp3 upregulation, which contributes to immune tolerance and suppression (86). IL-32α drives CD4+ T cell polarization into the Th17 phenotype by upregulating ROR-γt and increasing IL-17 production, which accelerates the inflammatory process (**Figure 3c**) (87). These findings illustrate the bifunctional role of IL-32, which either potentiates or suppresses the immune response according to the prevailing immunological milieu.

## 5 Cellular sources and induction of IL-32 expression by HIV

HIV infection has been shown to directly or indirectly induce the expression of IL-32. Consistently, an *in vitro* infection model using PBMCs from healthy volunteers with macrophage-tropic HIV strains resulted in the upregulation of multiple variants of IL-32, including (α, β, γ, ϵ, θ) as well as IL-32D. Among these isoforms, IL-32γ and IL-32D are the most significantly upregulated in PBMCs (26). Profiling of IL-32 transcription in PBMCs from HIV-positive participants demonstrated that T-cells and NK cells are major sources of most IL-32 isoforms, whereas IL-32γ is dominantly expressed in B lymphocytes and monocytes (88). HIV infection of Jurkat T cells has been found to remarkably promote IL-32 production compared with noninfected cells (89).

IL-32 has been observed to be persistently expressed in people living with HIV under antiretroviral therapy (ART). Mechanistically, HIV infection can mediate microbiota modification and translocation, which is significantly linked with increased IL-32 and TNF-α expression, as well as several other proinflammatory cytokines (26). TNF-α is a positive regulator of IL-32 in both immune and nonimmune cells (29, 35, 37).

The infection of intestinal epithelial cells (HT-29 cells) with HIV NL4.3BaL and THRO strains has been demonstrated to significantly enhance both transcription and translation of IL-32 (29). Altogether, HIV can stimulate IL-32 expression in both immune and nonimmune cells. However, the specific viral mediators and underlying molecular mechanisms orchestrating IL-32 expression have yet to be identified.

The rs4349147, a noncoding genetic variant which has been identified in the enhancer element of *IL-32* and shown to be significantly correlated with HIV-1 susceptibility (90). *In vitro* functional analysis of this variant in response to HIV challenge demonstrated that rs4349147 -/A Jurkat T cells exhibited alterations in chromatin conformation, accompanied by a significant reduction in IL-32 expression. However, rs4349147 G/− cells had higher IL-32 promoter activity, with robust upregulation of proinflammatory isoforms expression, including IL-32β/γ, compared with rs4349147 -/A cells. rs4349147 -/A shows significantly enhanced IL-32α transcription relative to non-α isoforms and decreased the susceptibility to HIV infection compared with the rs4349147 G/− (90).

## 6 Immunoregulatory functions of IL-32 during HIV infection

IL-32 plays a complementary role in HIV pathogenesis by shaping both protective immunity and immunosuppressive landscapes. IL-32 is also emerging as a strong contributor to cardiovascular disease, especially in chronically HIV-infected individuals.

### 6.1 IL-32 promotes host immunity to control HIV infection

IL-32 levels are significantly higher in the serum of asymptomatic people with HIV compared to healthy controls, suggesting that this cytokine may suppress viral replication. *In vitro* functional assays employing IL-32 silencing and ectopic expression in Jurkat T cell and HEK293 T cell lines demonstrated that IL-32 significantly attenuates HIV replication (**Table 1**). This is evidenced by the reduction by half of the HIV p24 concentrations in the supernatant of IL-32-expressing cells (89). The mechanisms of IL-32’s antiviral activity against HIV are yet to be elucidated. Ablation of IL-32 expression in PBMCs using RNA interference resulted in a pronounced reduction in proinflammatory mediators, such as IL-6, TNF-α, and IFN-γ, as well as helper T cell type 1 (Th1) mediators, accompanied by a significant increase in HIV-1 replication (91). Similarly, the silencing of IL-32 expression in macrophages also resulted in enhanced viral replication. In contrast, the treatment of PBMCs and macrophages with IL-32γ elevated IFN-α/β and attenuated HIV-1 amplification by 72% compared to control cells (**Table 1**) (91).

**Table 1:** Protective function of IL-32 during HIV infection.

Experimental condition	Key protective effects of IL-32	Reference
IL-32 overexpression	Suppresses HIV replication in Jurkat and HEK293 T cell lines.	89
IL-32 silencing	Limits the production of IL-6, TNF-α, and IFN-γ and promotes HIV replication in PBMCs and macrophages.	91
IL-32γ treatment	Increases APOBEC and MxA production and limits HIV replication in PBMCs	92
IL-32γ treatment	Inhibits HIV target receptor CD4 and coreceptor CCR5 expression in macrophages.	93
IL-32γ treatment	Inhibits viral reverse transcriptase in monocyte-derived macrophages.	21
IL-32γ treatment	Promotes IFN-α/β production and attenuates HIV replication in macrophages.	91
IL-32γ treatment	Augments viral replication in latently infected CD4+ T cells	88

APOBEC, apolipoprotein B mRNA editing catalytic polypeptide-like; MxA, myxovirus resistance protein A; PBMCs, peripheral blood mononuclear cells.

IL-32 is also markedly expressed in PBMCs from untreated people with chronic HIV-1 infection compared to cells from healthy controls. The increase in IL-32 transcription level was negatively associated with HIV-1 RNA levels, implicating an antiviral role for this cytokine. Mechanistically, IL-32γ stimulation of PBMCs upregulated the expression of IFN-stimulated genes, including *APOBEC* and *MxA* (92) (**Table 1**), both of which possess anti-HIV-1 properties (94, 95).

An ex vivo study of anti-HIV mechanisms of IL-32 revealed that IL-32γ stimulation markedly reduces the expression of the HIV entry receptor CD4 and co-receptor CCR5 on primary macrophages (**Table 1**), compared to unstimulated cells. As a result, IL-32γ impedes HIV-1 entry, restricts proviral DNA integration, and effectively suppresses viral replication (93). However, the precise pathway and molecular mediators by which IL-32γ blocks viral genome integration are yet to be determined. A recent study revealed that IL-32γ confers anti-HIV-1 activity at the post-entry stage in monocyte-derived macrophages (MDMs) via deregulation of cyclin-dependent kinases, thereby promoting the function of the viral reverse transcriptase inhibitor SAMHD-1. Notably, this anti-HIV effect is abrogated in cells following treatment with SAMHD-1-degrading protein, underscoring its crucial role in IL-32γ-mediated restriction of HIV replication (**Table 1**) (21). These findings indicate that IL-32γ exhibits anti-HIV effects by suppressing virus entry and replication, as well as promoting the synthesis of antiviral factors.

Six IL-32 variants, IL-32α, β, γ, ϵ, D, and θ, exhibit sustained and significant expression in PBMCs from HIV-infected individuals receiving combination antiretroviral therapy (ART), compared to healthy controls. Among immune cell subsets, these isoforms are expressed in CD4+ T cells and NK cells, with IL-32β being the predominant one (88). However, functional analyses showed that only IL-32γ augments viral replication in latently infected CD4+ T cells obtained from people with HIV receiving combination ART (**Table 1**) (88). This finding suggests that IL-32γ could be used in combination with ART to treat individuals infected with latent HIV. Understanding the specific mechanism by which IL-32γ mediates the reactivation of latent HIV may help in the development of an ART potentiator.

### 6.2 IL-32 promotes HIV immune evasion strategies

**Figure 4:**
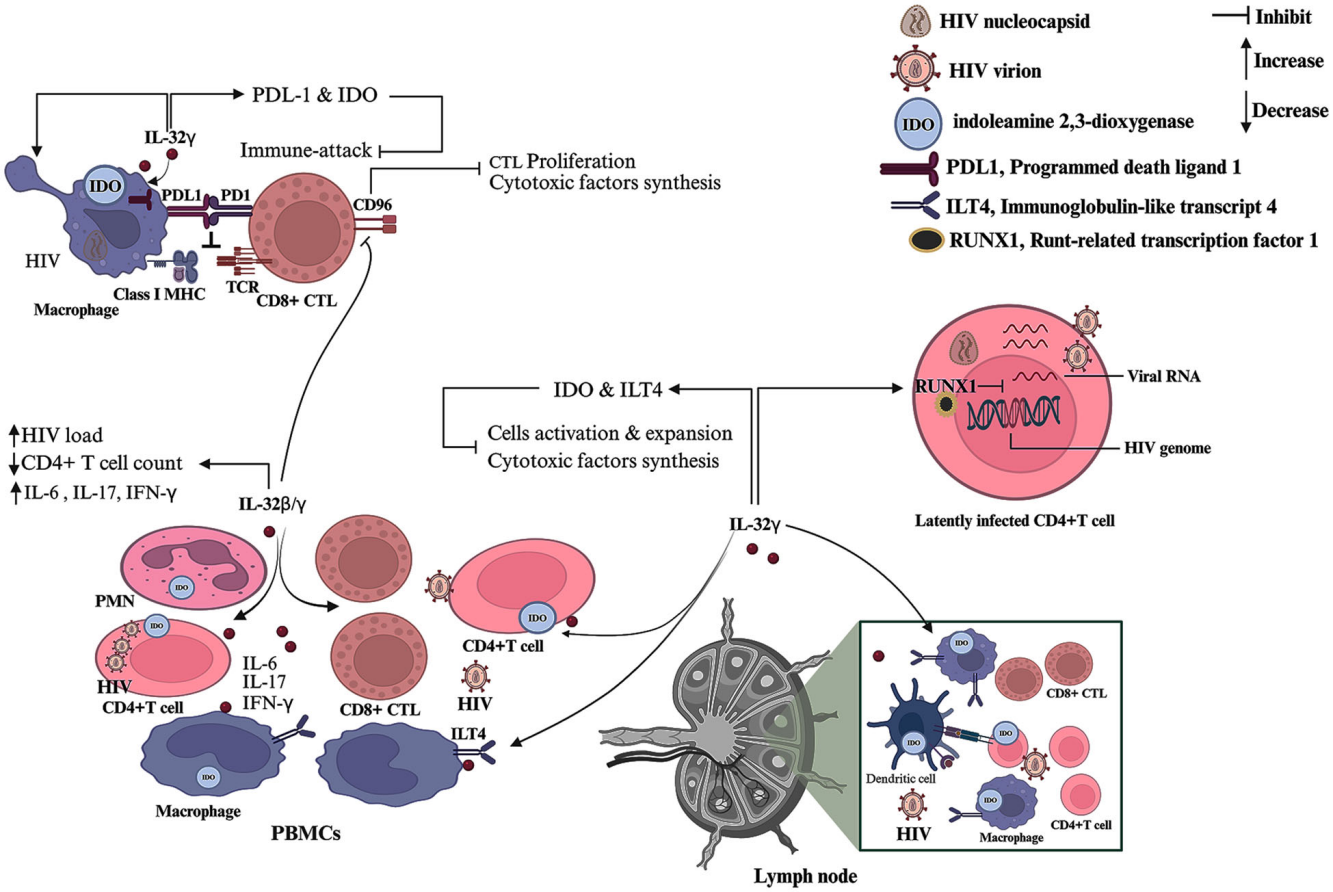
Modulation of host immune responses by IL-32 during HIV infection. IL-32γ induces the production of immunosuppressive factors, such as PDL-1 and IDO, by HIV-infected macrophages, thereby shielding these cells from cytotoxic immune attack. In addition, IL-32γ promotes the expression of IDO in a variety of immune cell populations in both peripheral blood and lymphoid tissues and induces the production of immunosuppressor receptor ILT4 in macrophages. The combined action of IDO and ILT4 inhibits host immune activity and diminishes the synthesis of cytotoxic mediators such as granzyme and perforin. Furthermore, IL-32γ facilitates the establishment of HIV latency in CD4+ T cells by increasing RUNX1 expression. IL-32β and IL-32γ exhibit dual functionality: they augment proinflammatory cytokine production, contributing to CD4+ T cell depletion and promoting viral replication; and they also impede CD96 expression, thereby restricting CD8+ T cell expansion and impairing cytotoxic mediator synthesis.

IL-32 can modulate host immune responses to promote HIV persistence and facilitate ongoing viral replication (**Figure 4**). IL-32γ has been shown to confer protection to HIV-1-infected MDMs against immune-mediated clearance and to augment viral infectivity by upregulating the production of the immunomodulatory molecules, indoleamine 2,3-dioxygenase (IDO) and programmed death-ligand-1 (PDL1). These inhibitory molecules are induced through the activation of NF-κB, p38 MAPK, and STAT3 pathways (21). IDO catalyzes the breakdown of tryptophan to kynurenic metabolites. This metabolic pathway exerts immunosuppressive effects by promoting the polarization and expansion of Treg cells, ultimately attenuating HIV-specific immune responses (96–98). Similarly, PDL1 impairs HIV-specific CD4+ and CD8+ T cell function, thereby enhancing viral persistence and replication (99). IL-32γ also facilitates the systemic dissemination of HIV-1 by enhancing the migration of infected MDMs by stimulating the development of invadopodium-like protrusions and diminishing cell adhesion capabilities (21).

Elevated serum levels of IL-32β and γ in untreated people with HIV infection are strongly linked to severe immunosuppression and heightened viral production. Immunological profiling has shown reduced CD4+ T cell counts and elevated levels of proinflammatory mediators, such as IL-6, IFN-γ, and IL-17, indicating that these isoforms may serve as potential prognostic markers for the disease (20). IL-32 has also been found to be markedly elevated in various cells, including CD4+ T cells, dendritic cells, macrophages, B lymphocytes, and gut epithelial cells, obtained from the lymphatic tissue and PBMCs of HIV infected people across all stages of the disease compared to healthy controls. Notably, IL-32 expression is significantly higher during both the acute and AIDS stages of infection compared to the asymptomatic stage (100), implicating a possible immunosuppressive function. Both ex vivo and *in vivo* experiments have shown that IL-32γ attenuates innate and protective immune responses; this finding is supported by diminished immune cell activation, limited expansion, and reduced secretion of cytotoxic mediators (e.g., perforin and granzyme) linked to increased HIV replication (100). The immunosuppressive activity of IL-32γ is mediated by increased expression of the immunomodulatory molecules, immunoglobulin-like transcript (ILT) 4 and IDO (100). An in-depth elucidation of the mechanisms underlying IL-32-induced upregulation of ILT4 and IDO could inform novel strategies for improved HIV management.

A primary obstacle in the treatment of HIV infection is the sequestration of the virus in CD4+ T cells as an integrated, non-replicative, silent provirus. Since ART targets the actively replicating virus (101). Therefore, deciphering strategies that induce the reactivation of latent infection may hasten viral clearance. IL-32 transcription is markedly increased in CD4+ T cells infected with latent HIV compared to both productively infected cells and naive cells, suggesting that it might be a potential biomarker for HIV latency (102). Interestingly, IL-32γ has been reported to suppress HIV reactivation in CD4+ T cells by inducing runt-related transcription factor 1 (RUNX1) (103). Therefore, the targeting of IL-32 signaling-mediated RUNX1 expression may improve the efficacy of ART.

A recent study demonstrated that IL-32β and γ diminish the proliferation and effector function of HIV-specific cytotoxic T lymphocytes through inhibition of CD96 expression, thereby facilitating disease progression (104). Furthermore, IL-32 can modulate CD4+ and CD8+ T cell subpopulations, as evidenced by its inverse correlation with naïve, effector, and memory T cell frequencies within the gut lamina propria (105), suggesting a potential manipulatory function for IL-32 in mucosal immunity.

### 6.3 IL-32 is a mediator of cardiovascular diseases in HIV

Cardiovascular disease (CVD) is the most predominant comorbid disease in individuals living with HIV and ultimately leads to the development of progressive heart failure. Proinflammatory cytokines are key players in HIV-mediated CVD (106). Although IL-32 is persistently produced in HIV-infected individuals, three isoforms, IL-32α, β, and ϵ, are noticeably upregulated in PBMCs obtained from women living with HIV who have atherosclerosis compared to those without. These findings suggest that these particular isoforms may be implicated in the pathogenesis of atherosclerosis (107). Similarly, the expression of six isoforms of IL-32, α, β, γ, ϵ, θ, and D, was significantly upregulated in HIV-positive men compared to healthy controls. Two of these isoforms, IL-32D and θ, were the most highly upregulated in HIV-infected individuals with coronary artery atherosclerosis (CAA), compared to those without CAA, which suggests CVD- and sex-specific IL-32 isoform expression. These two IL-32 variants were positively correlated with increased plasma concentrations of multiple proatherogenic mediators, including IL-1β, IL-18, vascular endothelial growth factor-A (VEGF-A), and fibroblast growth factor-23 (FGF-23), while showing a negative correlation with the anti-atherosclerotic protein TRAIL (26). However, the specific mechanisms of IL32D and θ involvement in the pathogenesis of atherosclerosis remain unclear. A recent study demonstrates that IL-32θ can orchestrate the polarization of M0 macrophages towards M1 phenotype through activation of the p38 MAPK and NF-κB signaling pathways, implicating its role in the inflammatory process (108). Besides, this isoform possesses both anti-inflammatory functions by suppressing IL-1β production (76) and anti-atherosclerogenic by attenuating monocyte adhesion to endothelial cells (84). Interestingly, ex vivo stimulation of monocytes with IL-32β and γ promotes their polarization toward the inflammatory macrophage (M1) phenotype. This phenotypic shift is accompanied by significantly increased production of proinflammatory mediators, such as IL-18 and IL-1β, which are implicated in atherogenesis, and dysregulation of the anti-atherogenic factor TRAIL (26).

IL-32β and γ can exacerbate the inflammatory reaction in the cardiac microenvironment by increasing IL-6 and decreasing IL-10 production by coronary artery endothelial cells (CAECs) (109). Both isoforms also markedly enhance the ability of CAECs to produce the monocyte chemotactic factors CCL-2 and CXCL-8, as well as the adhesion molecules ICAM-1 and VCAM-1. These increase the intensity of inflammation and endothelial cell dysfunction by monocyte infiltration. Clinically, IL-32 is significantly associated with the stiffening of the arteries, even in young individuals living with HIV (109), suggesting an increase in the probability of CVD occurrence. In contrast, an *in vitro* study showed that the new IL-32θ variant can attenuate TNF-α-induced upregulation of the adhesion molecules CD54 and CD106 in endothelial cells, thereby suppressing monocyte–endothelial cell adhesiveness, indicating that IL-32θ may represent a promising immunotherapeutic for atherosclerosis (84).

IL-32γ can drive the polarization of HIV-infected memory CD4+ T cells toward a cardiotropic phenotype, characterized by the upregulation of double chemokine receptors CCR4 and CXCR3, as well as receptor tyrosine kinase, which promotes the homing of these cells to cardiac tissue. Notably, in clinical observations, the frequency of these cells was considerably elevated in HIV-positive individuals with subclinical atherosclerosis than in those without vascular abnormalities (23).

Vascular calcification is characterized by the ectopic accumulation of calcium within the blood vessel wall, leading to loss of vascular plasticity, stenosis, and increased risk of CVD fatality (110). Recent evidence indicates a key role for IL-32 in this pathological process. IL-32β and γ polarize monocytes toward osteoblast-like cells, which are characterized by osteocalcin expression and strong capabilities to mediate calcium deposition. Supporting these findings, *in vivo* assessments of individuals with HIV have demonstrated elevated plasma levels of the vascular calcification biomarker osteoprotegerin, alongside detection of calcium plaques in coronary arteries, which is correlated with the presence of subclinical atherosclerosis (22).

## 7 Exploring IL-32 as a novel therapeutic target in HIV infection

**Figure 5:**
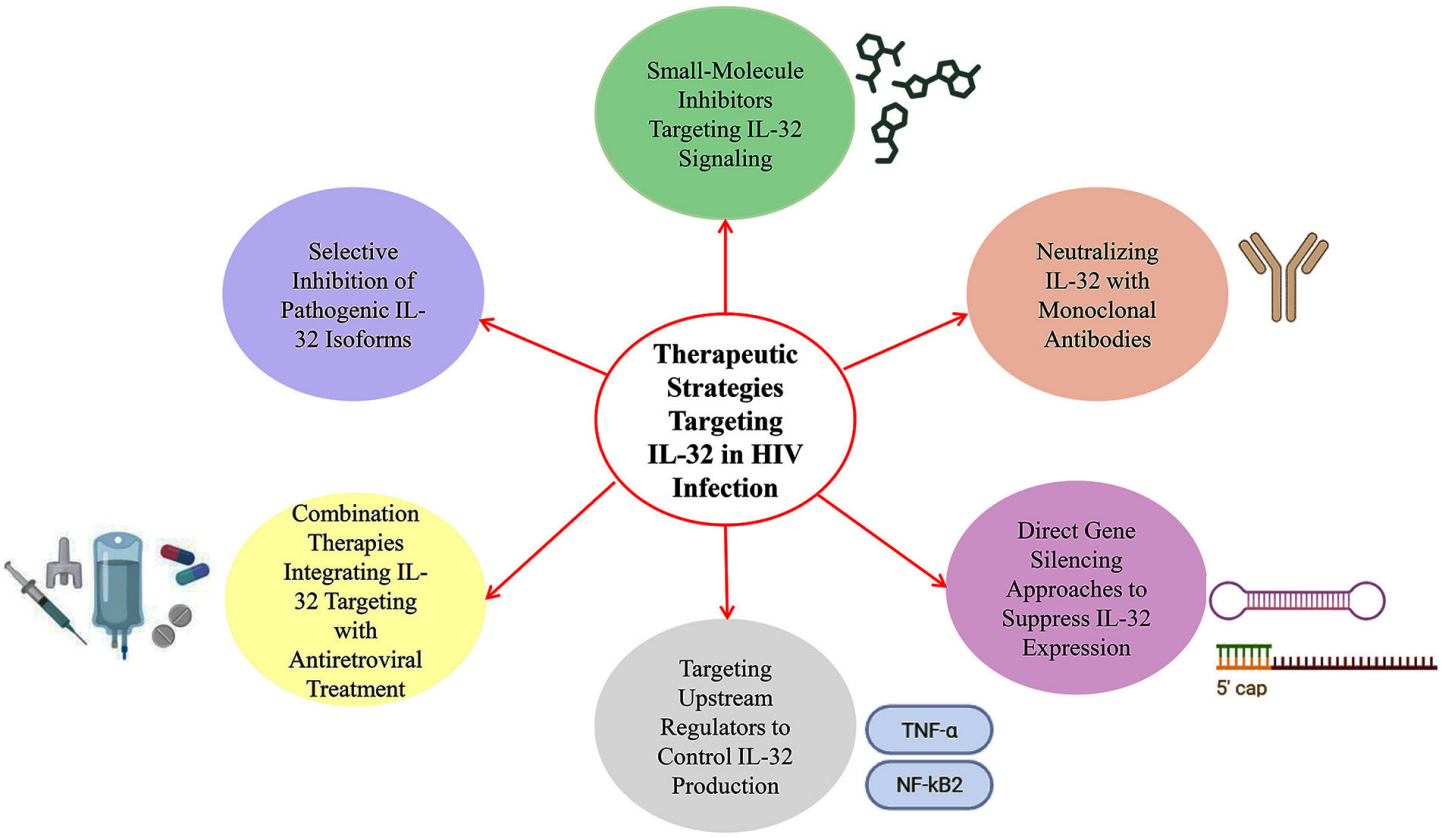
Emerging strategies for IL-32-targeted therapeutics.

IL-32 has been reported to play a central role in chronic immune activation and inflammation in HIV-infected individuals. Thus, the targeting of IL-32 could offer a novel therapeutic strategy to limit the exaggerated inflammatory response and immune dysfunction in people living with HIV. In this context, potential therapeutic strategies aimed at modulating IL-32 activity or expression are explored in this section. Currently, there are multiple directions in the development of IL-32-targeted drugs (**Figure 5**).

A potential therapeutic direction involves the design of small-molecule inhibitors that either specifically target IL-32 signaling pathways or block IL-32’s interaction with downstream effector molecules. They offer numerous advantages, including patient convenience, a lower risk of immunogenicity, suitability as an ideal option for optimizing drug properties, and relatively cost-effective production (111). Furthermore, small molecules have been shown to be highly successful in targeting interleukin-mediated pathways, offering precise modulation of immune responses and potential therapeutic benefits across a range of inflammatory, infectious, and autoimmune diseases (112, 113). However, the designing of inhibitors against IL-32 is challenging due to the presence of multiple isoforms with a wide range of biological activities and unidentified receptors or signaling mechanisms. Another avenue beyond small-molecule inhibitors is the use of antibody-based therapeutics, which represent one of the largest and most commercially important classes of biologics (114). Thus, the development of neutralizing monoclonal antibodies against IL-32 or its receptor, if characterized, offers a promising strategy to limit the IL-32–driven exaggerated inflammatory response in HIV-infected individuals, where its dysregulated expression contributes to chronic immune activation and associated comorbidities. This approach has been reported to be highly successful in targeting other interleukins, such as IL-6 and IL-17, indicating the therapeutic potential of neutralizing antibodies in modulating pathogenic IL-32 signaling (115, 116). Moreover, gene silencing technologies, such as small interfering RNA (siRNA) and antisense oligonucleotides, offer direct options to reduce IL-32 expression at the mRNA level (posttranslational level) (117, 118). These technologies can selectively decrease IL-32 production in immune cells involved in HIV pathogenesis, thereby limiting its proinflammatory effects. However, the successful delivery of these molecules to target cells *in vivo* remains a great challenge, alongside other challenges related to ensuring target specificity and minimizing off-target consequences. Recent advancements in the nanoparticle delivery area and viral vectors may enhance the feasibility of this strategy, enabling more precise modulation of IL-32 activity in individuals living with HIV. In addition to directly silencing IL-32 expression at the mRNA level using gene-targeting approaches, an alternative strategy is to intervene earlier in the regulatory cascade by targeting the upstream signaling pathways and transcription factors that govern its production. IL-32 expression is known to be induced by inflammatory stimuli such as TNF-α, mediated through transcription factors such as NF-κB. By inhibiting these upstream mediators, it may be possible to reduce IL-32 levels indirectly, thereby blocking the associated inflammatory events. Pharmacological inhibitors of NF-κB, such as BAY 11–7082 and MG132, have been shown to significantly downregulate IL-32 transcription and reduce its protein production (37, 38). Several of these inhibitors and other agents targeting inflammatory signaling pathways are already under investigation for various diseases, and repurposing or developing such compounds for HIV-associated inflammation could help manage IL-32-driven pathology in HIV-infected people (119, 120). Given the multifactorial nature of HIV infection and its associated immune activation, combination therapies that consist of IL-32-targeted interventions and standard ART may be the most effective strategy. While ART suppresses viral multiplication, it does not fully resolve chronic inflammation and immune activation. Combining ART with agents that reduce IL-32-mediated inflammation may synergistically improve clinical outcomes by addressing both viral and immunological factors. Careful evaluation of drug–drug interactions, safety, and efficacy will be essential for developing such combination regimens. This integrative approach has the potential to reduce HIV-associated comorbidities and improve the quality of life of people living with HIV. Building on the concept of integrating IL-32-targeted interventions into HIV treatment regimens, a more precise therapeutic approach would involve selectively inhibiting the IL-32 isoforms most strongly linked to pathogenic inflammation, while preserving those that exert protective or regulatory effects (88).

Despite the potential of the abovementioned strategies to improve the outcomes of patient infected with HIV through modulation of IL32, certain challenges could limit their usefulness in this regard. Thus, targeting IL-32 in HIV infection needs a carefully fine-tuned approach due to its multifaceted roles in immune regulation and inflammation. Although IL-32 has been reported to have antiviral capabilities, it can also trigger heightened pro-inflammatory responses that complicate HIV pathology. Therefore, therapeutic modulation must be selective to ensure enhanced viral suppression without exacerbating immune dysregulation. In this context, the biological complexity of IL-32 arises from the existence of multiple variants, each with unique functions. Moreover, a single isoform can regulate both pro- and anti-inflammatory mediators such as IL-32β (88). Besides, isoform-isoform interaction could result in opposing effects; for instance, IL-32β stimulates IL-10, whereas IL-32δ diminishes its release by sequestering IL-32β (121). Comprehensive isoform-interaction studies have disclosed several heterodimeric interactions amongst IL-32 variants, demonstrating that IL-32 activity is governed by a finely tuned network of isoform-isoform interactions rather than a single signaling cascade (25). Given these dynamics, advancing IL-32-based therapeutics in HIV infection necessitates rigorous investigation of individual isoform biology and interaction networks. Precision targeting designed to amplify beneficial antiviral functions, yet minimizing pro-inflammatory risks, will be essential in developing safer and more effective immunomodulatory strategies for better HIV control.

## 8 Conclusion and unresolved questions

IL-32 is a potent multifaceted immunomodulator with complex functions during HIV infection. Among its isoforms, IL-32γ is the most extensively investigated, with findings indicating that it can drive the host anti-HIV mechanisms to control viral infection through multiple mechanisms: (1) promotion of the production of anti-viral mediators and molecules such as IFNα/β, APOBEC, and MxA; (2) the blocking of HIV entry into the target cell by downregulating the viral target receptor and co-receptor; (3) suppression of viral reverse transcriptase; and (4) potentiation of ART activity by inducing the reactivation of latent viral infection. On the other hand, IL-32γ is associated with increased production of proinflammatory mediators, reduced CD4+ T cell count, and increased viral load. It can also provide HIV-infected cells with protection against immune clearance via the upregulation of PDL-1 expression in infected macrophages and downregulation of CD96 expression in CD8+ CTL, thereby reducing their capability to proliferate and synthesize cytotoxic molecules. IL-32γ also induces the expression of immunomodulatory molecules such as IDO and ILT4. These activities result in the expansion of Treg cells and ablation of innate and adaptive anti-HIV immune responses. This immunosuppressive state, alongside IL-32γ-mediated expression of RUNXI, contributes to viral persistence and establishment of latency within CD4+ T cells.

The persistent IL-32 production in people living with HIV is strongly linked to a high risk of CVD development. The underlying mechanisms have been investigated using IL-32β and γ isoforms. These isoforms exacerbate cardiovascular inflammation by inducing an imbalance in inflammatory mediators by (1) increasing the production of IL-6 and monocyte-chemotactic factors CCL-2 and CXCL-8; and (2) reducing the secretion of the anti-inflammatory mediator IL-10 by CAECs. Furthermore, IL-32β and IL-32γ drive the polarization of monocytes into inflammatory cells with atherogenic properties, characterized by the secretion of pro-atherogenic factors and the ability to promote vascular calcification. IL-32γ contributes to the development of coronary atherosclerosis by stimulating resident HIV-infected CD4+ memory T cells within cardiac tissue.

Despite the accumulating evidence gained in deciphering IL-32 biology and its role during HIV infection, several key questions remain unanswered. Which cells are the principal source of IL-32 during HIV infection? What are the molecular pathways, specifically receptors, transcription factors, and their cascade, which govern the expression of different IL-32 isoforms in response to HIV infection? How can novel detection approaches be developed to accurately identify the mRNA and protein expression patterns of its distinct isoforms? How do *IL32* gene mutations at the promoter region and splicing site influence isoform diversity and alter the immune response of HIV-infected individuals? Much of the literature focuses on IL-32γ, with few examining IL-32β; therefore, the obvious question remains: How do other isoforms, such as IL-32θ, contribute to inflammatory and immunosuppressive processes during HIV infection?

It is unclear how the balance between IL-32’s pro- and anti-inflammatory effects is regulated spatially and temporally during different phases of HIV infection and in various tissues. A considerable challenge is the absence of the *IL32* gene in HIV-susceptible animals (122). IL-32 transgenic nonhuman primates would be invaluable for gaining an in-depth understanding of the role of this cytokine in HIV infection. Finally, an understanding of how IL-32 regulates HIV reactivation and latency will undoubtedly be helpful for the development of therapeutic adjuvants.
